# Temporal Feeding Pattern May Influence Reproduction Efficiency, the Example of Breeding Mares

**DOI:** 10.1371/journal.pone.0073858

**Published:** 2013-09-30

**Authors:** Haifa Benhajali, Mohammed Ezzaouia, Christophe Lunel, Faouzia Charfi, Martine Hausberger

**Affiliations:** 1 UMR CNRS 6552 Ethologie Animale et Humaine, Université de Rennes I, Rennes, France; 2 Haras national de Sidi Thabet, Sidi Thabet, Tunisia; 3 Unité de Biologie Animale et de Systématique Evolutive, Université Tunis-ElManar, Campus universitaire, Tunis, Tunisia; University of Sao Paulo, Brazil

## Abstract

Discomfort in farm animals may be induced by inappropriate types or timing of food supplies. Thus, time restriction of meals and lack of roughage have been shown to be one source of emergence of oral stereotypies and abnormal behaviour in horses which have evolved to eat high-fibre diets in small amounts over long periods of time. This feeding pattern is often altered in domestic environment where horses are often fed low fibre meals that can be rapidly consumed. This study aimed at determining the effect of the temporal pattern of feeding on reproductive efficiency of breeding mares, One hundred Arab breeding mares were divided into two groups that differed only in the temporal pattern of roughage availability: only at night for the standard feeding pattern group (SFP mares), night and day for the “continuous feeding” group (CF mares). The total amount of roughage provided was the same as the CF mares received half of the hay during the day while in paddock (haynets). Mares were tested for oestrus detection by teasing with one stallion and were then examined clinically by rectal palpations and ultrasound before being mated naturally or inseminated by fresh or frozen semen. Multivariate logistic regression was used to analyse data. The treatment affected significantly the reproductive efficiency of the mares with fewer oestrus abnormalities (*p* = 0.0002) and more fertility (*p* = 0.024) in CF mares (conception rate = 81% versus 55% in SFP mares). Ensuring semi-continous feeding by providing roughage may be a way of fulfilling the basic physiological needs of the horses' digestive system, reducing stress and associated inhibitors of reproduction. To our knowledge, this study provides the first evidence of an impact of temporal feeding patterns on reproductive success in a Mammal. Temporal patterns of feeding may be a major and underestimated factor in breeding.

## Introduction

Many field observations and induced-stress studies showed that at least acute stress events may impair reproduction efficiency in sheep [1], [2], [3], [4], cattle [5], rhesus monkey [6], [7], rodents [8], [9], [10] and human [11], [12]. In fact, activation of the hypothalamus–pituitary–adrenal axis by stressors reduces the pulsatility of GnRH/LH by actions at both the hypothalamus and pituitary gland, ultimately depriving the ovarian follicle of adequate LH support. This may lead to reduced oestradiol production by slower growing follicles [13]. However, effect of stress on reproduction may involve factors other than hormones. Davis and Cole (1943, In [14]) found that decreased concentrations of ascorbic acid that have been later associated with stress [15] reduce breeding efficiency in the mare.

Adverse effects on reproductive efficiency have been observed after exposure to different types of management stress (acute or chronic) in sheep, cattle and pigs (transport: [5], [16], [6]; insulin administration: [3]; cold stress: [17]; tethering/individual housing: [18], [19]; social stress: [20]) during the follicular phase.

Chronic stress may also been induced by inappropriate types or timing of food supplies. Thus, time restriction of meals and lack of roughage have been shown to be one source of emergence of oral stereotypies and abnormal behaviour in horses [21], [22], [23]. Gastric ulcers are frequent in domestic horses [24] which may be due to the lack of roughage and the time spent with an empty stomach [25]. Gastric discomfort may occur if the stomach is empty for one or two hours in this species [26] due to a digestive system adapted to foraging for long hours on high fibre diets [27]. Horses are “trickle feeders” [26] which require a semi-continuous supply of small amounts of forage. In a natural environment, they spent 40 to 70% a day foraging and disperse this behaviour throughout the entire 24-h period although most time feeding is during daylight (outside hot summer days) (e.g. [28], [29], [30], [31]. In a previous study we could show that semi-continuous feeding provided an increased welfare with a more usual time budget, an enriched behavioural repertoire characterized by more occurrence of “relaxed” behaviour (e.g. lying down) and more positive social interactions [32]. Previous studies have shown that inadequate nutrition or body condition had major effects on different breeding parameters in mares [33], [34], but the processes involved are not known [35]. In different species of birds, duration and time of food availability have been shown to influence males' gonadic growth [36].

Therefore we hypothesized that providing semi-continuous feeding to breeding mares may also improve their reproduction efficiency by in particular reducing the discomfort of time limited feeding opportunities. Two groups of mares were constituted that differed only by the feeding time schedule: hay at night for the standard feeding pattern group (SFP mares), hay at night and day for the continuous feeding mares (CF mares), while the total amount provided was kept alike. As mares were in group in a bare paddock during the day, hay was provided in the CF group through haynets hung at the fences (same number as the number of mares).

## Materials and Methods

### Animals and Study Site

The experiment was conducted between the 1^st^ April and 11^th^ June 2006 at the national breeding facility of Sidi Thabet, located at 20 km from Tunis, Tunisia (Latitude: 36° 20′ 6′′ N; daylength: 13–15 hours). Mares are brought to this facility every year in order to be bred with the stallions housed there. They were housed in individual boxes at night where they received barley grains (4kg per day) and hay (10 kg per day, see further) every morning and evening (4 p.m.). They were released every day from 9 a.m. to 3 p.m. in a paddock where free access to water and limited shelter (5 trees) were provided. No food was available then but some freshly cut grass was left on the ground around 12 a.m. every day and was entirely consumed by mares within an hour. Temperatures ranged from 7 to 40°C during the experiment (see also [32], [37].

We used 100 purebred Arab mares, aged 4 to 21 years (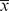 = 8.49±4.96). Mares had been present at the stud for 1 week at least and 3 weeks at most. They were housed all the time in individual boxes before the experiment. None had been bred for the ongoing season on the 1^st^ April 2006. A plastic name tag attached to a collar was used for the identification of each mare. All the mares were clinically healthy, in good body conditions and reproductively sound at the beginning of the experiment.

### Experimental procedure

The 100 mares were randomly divided into two groups:

In the continuous feeding group (CF) (n = 50), 50 haynets were hung in the paddock and filled with 5 kg each every morning before the arrival of the mares. Haynets were placed 3 m apart. Therefore they had 6 hours to eat the 5 kgs (which they did) during the day and 17 hours to eat the barley and remaining 5 kgs hay when in box.
In the standard feeding pattern group (SFP) (*n* = 50), no hay was provided in the paddock. Therefore they had 7 hours to eat the 10 kgs at night.

Both groups were kept in similar (4350 m^2^) bare paddocks, that is at the same density (115 mares/ha). All the mares were kept in individual boxes for the night under the same management conditions. However, the CF mares received 5 kgs hay only in the evening while in the box, so that both standard feeding pattern and experimental animals had access to the same total amount of food and differed only in their temporal distribution (6 hours more access to hay for the CF mares).

### Ethical point

The horses' owners gave full permission to the Director of the facility, Dr Ezzaouia, to proceed to any study including this one. Dr Ezzaouia is co-author of the manuscript. All management issues of the horses were under control of the facility, the high density of animals is usual in the facility and was not designed for the research purposes. The study is in agreement with current French laws (Centre National de la Recherche Scientifique) related to animal experimentation and were in accordance to the European Communities Council Directives of 24 November 1986 (86/609/EEC).

### Reproduction management and data collection

Oestrus was detected once every 48 hours by teasing with a stallion in early morning. Mares detected in oestrus were examined then clinically by rectal palpation and ultrasound to verify the follicular status of the mare. Matings or inseminations were performed at 48 h-intervals until ovulation was detected. Pregnancy detection was performed by ultrasound examination at days 15 post ovulation. Mares diagnosed pregnant were re-examined at days 30 post ovulation to confirm pregnancy. Teasing and matings were performed by a single team of three people. Rectal palpation, artificial insemination and pregnancy diagnosis were made by a single veterinarian. No medication or hormonal treatment was used for these mares.

Teasing, ultrasound examinations, veterinary treatments, artificial inseminations and matings were monitored for all the mares. For each ultrasound examination, the follicular and the uterus status of the mare were noted. Were excluded from this analysis the breeding data of the mares which were not clinically healthy (lameness, sickness, injury during the stay…), which left the stud before they had been diagnosed for pregnancy (owner's decision) and those which were not bred further due to the decision of a new owner (sold before breeding diagnosis). Breeding data for 102 oestrus cycles corresponding to 32 experimental mares and 38 standard feeding pattern mares were thus kept for statistical analysis. In total, 32 mares were pregnant after the first service, and therefore only 35 were submitted to a second (or more) service (3 SFP mares failed to show oestrus and were thus not bred). Mares were bred with 9 stallions housed in the facility, 55 were mated naturally and 12 inseminated with fresh semen according to the owner's decision (Table 1). The type of mating and stallions distributions were balanced between the two groups of mares (χ^2^, *p*>0.05 in both cases). The fertility per group of mares was determined by two parameters: the overall fertility rate (the percentage of pregnant mares at the end of the experiment) and the first-service fertility rate (the percentage of pregnant mares at the first service).

**Table 1:** Age and type of mating of the experimental (*N* = 32) and the standard feeding pattern (*N* = 38) groups.

	*Age (mean ± S.D.)*	*N.M.* 1	*A.I.F.* 2	*Not mated*
Experimental group	8.0±5.4	24	8	0
Standard feeding pattern group	7.8±4.4	31	4	3
Total	7.8±4.8	55	12	3

1 Natural mating.

2 Artificial insemination with fresh semen.

### Statistical analysis

Statistical analyses were conducted using the computer statistical program, *Statistica (v. 6.1, Statsoft, Tulsa, Oklahoma, USA)*. All values are given as means ± S.E.

For each mare mated or inseminated three variables were created:

OF (Overall Fertility): a bimodal variable which takes the value 1 if the mare was diagnosed pregnant at the end of the experiment and 0 if it was not.
FF (First-service fertility): a bimodal variable which takes the value 1 if the mare was diagnosed pregnant at the first service and 0 if it was not.
EA (Oestrus Abnormalities): a bimodal variable which takes the value 1 if the mare showed oestrus abnormalities (silent heat, prolonged diestrus) during the experiment and 0 if it was not.

We used a multivariate logistic regression, to analyse breeding data. The model for analysis of factors for the overall and first-service fertility included the mare's age, the stallion (S), the type of mating (assisted natural mating or artificial insemination, TM) and the hay-treatment (HT). The model for analysis of factors for the oestrus abnormalities included only the mare's age and the hay-treatment. We chose logit link function, binomial distribution and we used log-likelihood-ratio (*LL*) and Wald tests (*χ^2^ Wald*) to test the factors' effects. Since only some of the mares had a second service we were not be able to test for repeated measures. Additional χ^2^ tests have been performed when appropriate (proportion of mares successfully mated).

### Competing interests

The “Haras National of Sidi Thabet” made its horses available for the present study but had no role in study design, data collection and analysis, decision to publish or preparation of the manuscript. This does not alter our adherence to all the PLOS ONE policies on sharing data and materials. Dr Ezzaouia was affiliated to this company. This does not alter our adherence to all the PLOS ONE policies on sharing data and materials.

## Results

### Oestrus abnormalities' frequency

Only 2 CF mares versus 16 SFP mares showed oestrus abnormalities. The logistic regression results showed that the hay-treatment affected significantly the frequency of oestrus abnormalities (*LL* = −37.91, *χ^2^*  = 13.91, *p* = 0.0002). There was no significant effect of the mare's age (*LL*  = −32.33, *χ^2^*  = 2.76, *p* = 0.096) on the frequency of oestrus abnormalities. Because mares that failed to show oestrus were not always clinically examined to verify their follicular status, it was not always possible in all the cases to distinguish silent heats from prolonged diestrus.

### The first service fertility

**Figure 1:**
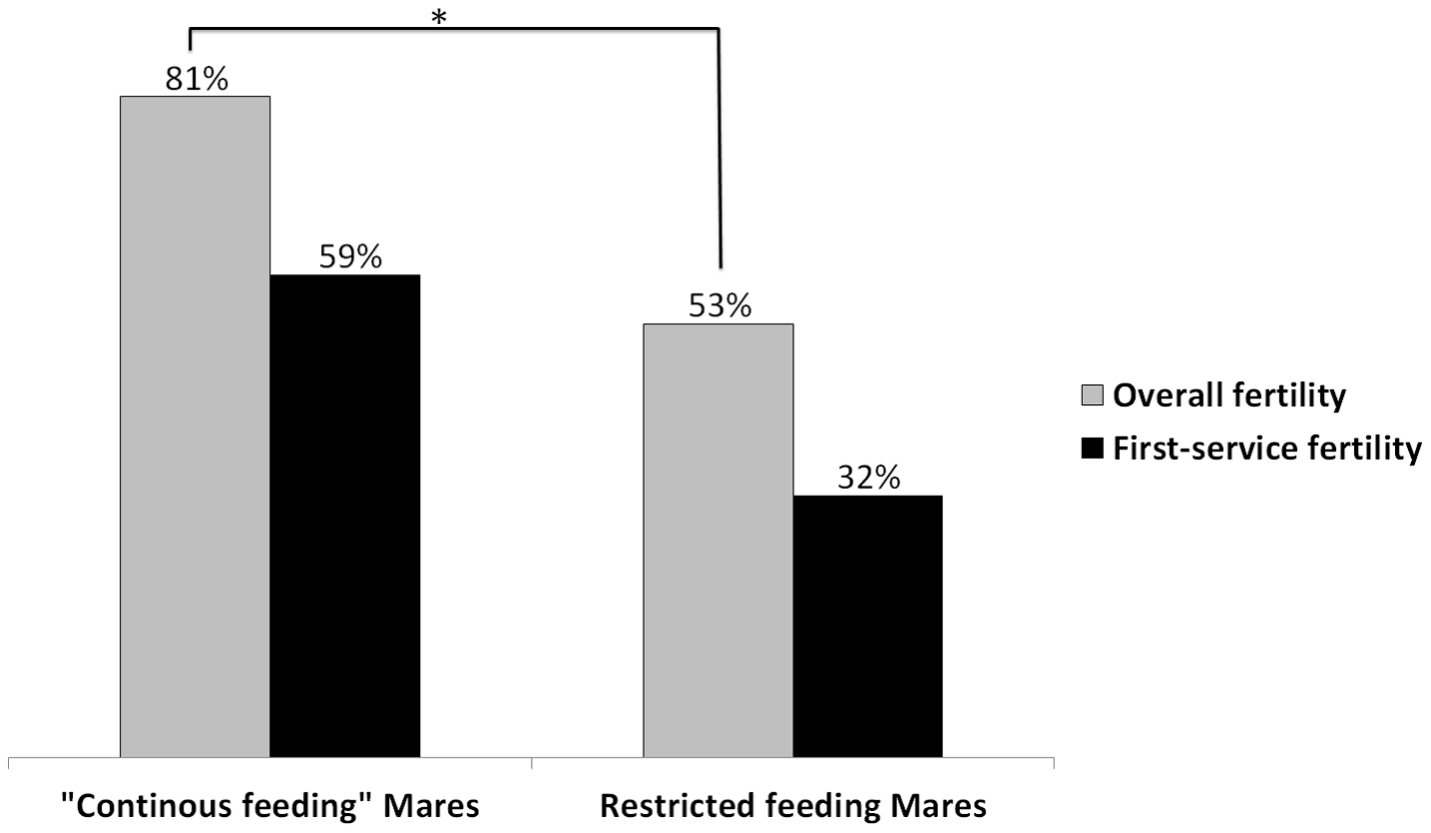
First-service and overall fertility (%) of CF (*N* = 32) and RF (*N* = 38) mares. significant differences (*p*<0.05) are indicated by the symbol *.

The first-service fertility of the CF mares was higher (59% *vs*. 32%, *LL*  = −37.65, χ^2^  = 2.45, p = 0.12) than in the SFP mares (see Fig. 1) which was confirmed by a chi square test (χ^2^ = 6.693, p = 0.01). None of the other factors (age of the mare, stallion, type of mating and all possible interactions) affected significantly the first-service fertility (all *p* values >0.2).

### The overall fertility

The logistic regression results show that the hay-treatment affected significantly the overall fertility of the mares (81% *vs*. 53%, *LL*  = −34.58, *χ^2^*  = 5.08, *p* = 0.02) (see Fig. 1). Moreover, while no difference was observable between the groups before the experiment, it became obvious for experimenters and caretakers at the end of the experiment that the continuous feeding mares had improved their body condition. A body condition score (INRA 1997) was applied and revealed a much better condition in the CF females (BCS  = 3,01±0.1 on a 5 point scale) than in the SFP mares (BCS = 2.57±0.1) (*p*<0.01).

## Discussion

The results obtained in the present study reveal that the mere temporal pattern of feeding, may have a major impact on reproductive success in horses. Thus a “continuous foraging” pattern decreased oestrus abnormalities and increased conception rates. Coming closer to the natural patterning of foraging behaviour in horses may be thus an easy and efficient way of increasing reproduction in the domestic situation.

Although it was not possible to duplicate groups in this study, the strong effects observed are not likely to be mere group effects. Such findings open also the line for applied issues on the management of domestic and captive animals. It is crucial to consider that regulating the timing of feeding may be more important than the amount of food provided per se.

The results converge with other data showing a general improvement of welfare, as revealed by behavioural indicators, under such regimen in horses [32], [38]. They are also in agreement with the current knowledge on horses' physiological system [26]. The precise mechanisms involved remain however to be discovered. One hypothesis is that the usual practice where horses are given limited amounts of roughage in a limited time span is stress inducing discomfort [37] and that the resulting chronic stress may affect reproduction in horses as it does in pigs [18], sheep [3], [39] and cattle [5]. The effect of the stress on reproductive disorders has been reviewed by Dobson and Smith [13] and the adverse effects of stress on both oestrus expression and the maintenance of pregnancy in several species are clear. ACTH and endogenous corticosteroids have been shown to interfere with oestrus behaviour and can both shorten and delay the onset of oestrus and interfere with hormonal events around ovulation [13]. Stress was shown to suppress oestrus behaviour in several mammals like rats [40], sows [41], ewes [39] and dairy cows [42]. However, the literature on the effect of mares' management on their reproductive performances is scarce. Stress caused by transportation does not seem to lead to fertility problems [14], [43]. The management of mares in relation to artificial insemination (gynaecological examinations together with transportation and loss of social partners) may act as a stressor and induce greater concentrations of cortisol secretion but no effect of cortisol on fertility parameters (oestrus duration, pregnancy rates) has been found in the study of Berghold et al. [44]. The possible reasons for these equivocal results are at least two-fold.

Firstly, it has been shown that the stress response depends on the intensity and duration of the stressor [13]. The stress-induced lows GnRH/LH pulse frequencies in proportion to the intensity of the stressor. In the case of a chronic stress of more severe lameness or fever, the pulse GnRH/LH frequency will be so slow that initial follicular growth will occur but will be unable to continue in to the later stages that depend on faster pulse frequencies. Thus, the animal fails to maintain oestrus cycles. However, in slightly less stressful situations, GnRH/LH pulse frequency may be just fast enough to support follicular growth and oestrus and fertilisation may occur [13]. The increase in cortisol secretion seen in the study of Berghold et al. [44] which was relatively low compared to the situation in horses after exposure to pain related to abdominal distress or castration [45] was probably insufficiently intense to produce a negative effect on mares' fertility. In the same direction, lack of impact of 12h transportation on mares' fertility [14] may be due to the relatively short stress duration (once for 12h) or to the preovulatory stage of the mares tested as like in ewes [46], [47], stress reaction was shown to decrease during oestrus in the mare [48], [49].

Subjective perception of the situation by the animals is another important factor. Purebred Arab horses used in this experiment are known for their high emotional reactivity [50] which is often considered as a variable modulating the behavioural and physiological responses to a negative situation [51]. Thus the high emotional reactivity of Arab horses used in the present study may intensify their response to the inappropriate environmental conditions.

A second alternative or additional hypothesis is that continuous feeding has an influence on the mares' metabolism and hence its effect on reproduction is mediated by body weight/condition observed in this study.

There are two reasons why this may happen here: 1) deprived mares (no hay net) spend more time in active locomotion [32], [37] and therefore have a much higher energy expenditure. This is in agreement with Piccione et al. 's findings [52] that feeding behaviour influences locomotor activity in horses, 2) as continuous feeding is in adequation with the digestive system of horses, these mares had less chances to experience gastric discomfort and potential digestive disorders. By triggering digestion, CF may have led to the observed enhanced body condition.

The treatment may have influenced the reproduction performances of the mares via an effect on body condition as leptin, a protein synthesised and secreted by the adipose tissue which acts as a signal to the central nervous system as an indicator of energy stored in adipose tissue appears to modulate the neuroendocrine system and the regulation of reproductive processes [53]. This hormone may be involved with the stimulation of the hypothalamus–pituitary function, as recently suggested for sheep [54], cattle [55] and horses [56]. This hormone has been identified in horses and circulating plasma concentrations increase as adiposity increases [57]. As hormonal analyses could not be performed in this study, further studies are needed to test further this hypothesis.

Nevertheless the results are in accordance with earlier reports showing that inadequate nutrition or lower body condition may decrease pregnancy rate and fertility overall [35]. A decrease in endogenous leptin secretions can be associated with the cessation of reproductive activity during the anovulatory season in mares [58]. The processes involved are poorly known overall [35]. One line of explanation may come from birds'studies showing that ad libitum feeding is the best regimen to induce testes' growth in blackheaded buntings (*Emberiza melanocyphala*) or house sparrows (*Passer domesticus*) [36]. But thesestudies also reveal that both duration and timing of food availability are important as synchrony between light and food cues seem to be critical. Food would influence the endogenuous circadian clock regulating reproduction.

In our study, the SFP mares had no food left in the morning, when horses would feed most in natural conditions. Therefore both the duration (6 hours more with roughage) and timing (feeding opportunities from 9 am to 3 pm) of feeding may explain the increased reproductive success in the continuous feeding mares.

To our knowledge, this is the first study showing the effect of temporal pattern per se of feeding on fertility in a Mammal. The findings that fertility may be improved by simple modifications in the timing of food distribution may have important implications and deserve further consideration to elucidate the processes involved.
